# Insomnia symptoms and related factors in a community-based population: The Yamagata Cohort study

**DOI:** 10.1016/j.heliyon.2024.e28228

**Published:** 2024-03-15

**Authors:** Miho Suzuki, Natsuko Suzuki, Kaori Sakurada, Norihiko Tsuchiya, Yoshiyuki Ueno, Tsuneo Konta

**Affiliations:** a Department of Public Health and Hygiene, Yamagata University Graduate School of Medical Science, Yamagata, Japan; b Department of Health and Nutrition, Yamagata Prefectural Yonezawa University of Nutrition Sciences, Yonezawa, Japan; c Department of Fundamental Nursing, Yamagata University Graduate School of Nursing, Yamagata, Japan; d Department of Urology, Yamagata University Faculty of Medicine, Yamagata, Japan; e Department of Gastroenterology, Yamagata University Faculty of Medicine,Yamagata,Japan

**Keywords:** Insomnia, Community residents, Cohort study

## Abstract

**Objective/background:**

Insomnia is prevalent and is a risk factor for the development of lifestyle-related diseases and early death. To improve insomnia, it is necessary to identify the factors that affect it. This study investigated the associations between insomnia symptoms and mental, physical, and environmental factors in the general Japanese population.

**Patients/methods:**

The study participants were 7,873 individuals who responded to the Health and Lifestyle Survey questionnaire that included sleep-related items between December 2021 and March 2022. Insomnia symptoms were defined as a score of 6 or higher on the Athens Insomnia Scale (AIS). A multivariate logistic regression analysis was performed to identify factors independently associated with insomnia symptoms.

**Results:**

Of all subjects, 23.4% had insomnia symptoms. Factors associated with insomnia symptoms were older age, female sex, very difficult living conditions on current income, pain/discomfort, anxiety, lack of happiness, frequent nocturia, long duration from bathing time to bedtime, bedroom lighting, and short walking duration. The subgroup analysis showed stronger associations between walking time in men, higher body mass index in women, time from bathing time to bedtime, and daily walking duration in older adults.

**Conclusions:**

Insomnia symptoms were common in community-based populations and were independently associated with three different factor groups including physical, psychological, and environmental factors. Improvements in insomnia symptoms require appropriate practical support tailored to an individual's situation.

## 1 Introduction

Insomnia, in terms of less duration or deterioration in sleep, is associated with an increased risk of lifestyle-related diseases, such as cardiovascular disease [1], hypertension [2], type II diabetes [3], and premature death [4]. In 2014, the Guidelines for Sleep for Health Promotion proposed lifestyle and environmental goals for good sleep [5]. However, the 2021 Health Survey by the Japanese Ministry of Health, Labor and Welfare showed that sleep problems were prevalent, such as waking up during sleep (44.6%), feeling sleepy during the daytime (37.2%), and dissatisfaction with the overall quality of sleep (34.7%), indicating that ensuring an adequate quantity and quality of sleep is an urgent issue in Japan [6].

Sleep is influenced by various factors, including pain, anxiety, nocturia [7], well-being [8], bedtime, and food intake [9]. Factors associated with sleep may vary by region and culture. In Japan, the previous studies reported the association between sleep status and various factors, including physical factors (diet quality [10], older age, lack of exercise [11]), psychological factors (psychological stress, perceived health [11], anxiety/depression [12], mental health problems [13], subjective happiness [14], health-related quality of life [15]), and environmental factors (unemployment [11,13], less years of schooling for women [13], household expenditure [14]). However, these reports mostly examined associations with one or two of the three different factor groups (physical, psychological, and environmental) or small numbers of factors. Since all three factor groups are related to sleep status, it is necessary to include these broad factor groups simultaneously to clarify the factors associated with sleep. In addition, although sleep status varies by age and gender, the analysis for these subgroups has not been adequately examined.

In this study, we examined the independent associations between sleep and a wide range of factors consisting of three different factor groups, both in the overall population and in subgroups by gender and age using data from the Yamagata Cohort Study, which contained information on sleep status and various background factors.

## 2 Methods

### 2.1 Study participants and survey procedures

The Yamagata Cohort Study is a prospective study supported by the 21st Century COE Program and the Global COE Program and was conducted in seven cities in Yamagata Prefecture (Yamagata, Sakata, Kaminoyama, Sagae, Higashine, Tendo, and Yonezawa). The study participants were a community-based population covered by the National Health Insurance system and comprising workers in the agriculture, forestry, and fishery industries who were self-employed, part-time employed, retired, and unemployed. There were 20,969 study participants at baseline (2009–2015) in this study. After excluding 3,442 subjects who had died or moved away by December 2021, Health and lifestyle questionnaires were mailed to the remaining 17,527 participants from December 2021 to March 2022. The completed questionnaires were returned by 12,216 participants (69.7% response rate). After excluding 4,343 participants with missing information on sleep status and background factors, we analyzed the questionnaires of 7,873 participants (3,304 men, 4,569 women) with valid sleep status responses.

### 2.2 Study items

A wide range of information was collected on personal characteristics, including physical factors (age, sex, body mass index [BMI], pain/discomfort, and nocturnal urination frequency), psychological factors (anxiety and happiness), and living conditions (daily walking time, time between bathing time and bedtime, bedroom lighting, time spent using smartphones and other devices, and living status on their current income) that may be related to sleep.

There are two questions to assess physical factors. Q1 ″Do you have pain/discomfort?" with answers to the five options (no pain/discomfort, some, moderate, considerable, extreme). Considering the bias in the number of responses, the answer "no pain/discomfort" was classified as "No" and the rest as "Yes". Q2 ″ How many times during the night did you get up to urinate?" with the answers to the four options (0 times, 1 time, 2 times, 3 times).

There are two questions to assess psychological factors. Q3 ″Do you feel anxious?" with the answers to the five options (not anxious, a little, moderately, considerably, extreme). Q4 ″How happy do you feel?" with the answers to five options (very happy, fairly happy, neutral, not very happy, not happy). Considering the bias in the number of responses, the answers "not anxious" to Q3 were classified as "No" and the rest as "Yes. For Q4, "very happy," and "fairly happy," were classified as “Yes”, and the rest as “No”.

There are five questions to assess environmental factors. Q5 “How many minutes do you walk in a day?” We categorized the walking time into four groups (<30 min, <1 hour, <2 hours, ≥2 hours). Q6 “How is life with your current income?” with the answers to the five options (very comfortable, somewhat comfortable, neutral, somewhat difficult, very difficult). Q7 “How long is the time from bathing and bedtime (min)?” with the answers to the five options (<60 min, 60–89 min, 90–119 min, 120–149 min, ≥150 min). Considering the bias in the number of responses, we categorized the obtained five-level responses into three groups (<60 min, 60–119 min, ≥120 min). Q8 “How is the bedroom lit while you sleep?” with the answers to the four options (no lighting, slightly lighting, lighting, others [free comments]). Q9 “How many hours per day do you spend on your smartphone, computers, and tablets combined?” with the answers to the five options (no use, <1 hour, 1–2 hours, 3–4 hours, ≥4 hours).

To examine the presence of insomnia symptoms among community residents, we used the Athens Insomnia Scale (AIS), a widely used self-report measure on the International Classification of Diseases, Tenth Edition (ICD-10). The AIS questions included eight items: time to fall asleep, mid-afternoon awakenings, early morning awakenings, sleep duration, sleep quality, daytime mood, physical and mental activities during the day, and daytime sleepiness [16]. Scores range from 0 to 24, and a score of 6 or higher is considered insomnia [17]. A recent reliability generalizability meta-analysis, including studies in Japanese subjects, have shown that the AIS has excellent internal consistency and retest reliability [18]. The Japanese version of the AIS has also been reported to have adequate validity and diagnostic utility [19]. In a study examining the validity of the Japanese version of the AIS for pathological insomnia in Japanese, the cutoff value was 5.5 points [19]. Based on this report, a score of 6 or higher was considered as having insomnia symptoms in the present study.

### 2.3 Statistical analysis

Student's t-test and chi-square test were used to examine the differences in continuous and categorical variables between those with and without insomnia symptoms. Simple and multiple logistic regression analyses were performed to identify factors independently associated with insomnia symptoms. The software JMP Pro16 for Windows were used for these analyses. The fitness of the multivariate regression analysis was checked using the Hosmer-Lemeshow goodness of fit test by R-based free software EZR version 2.8.0 (Jichi Medical School, Saitama, Japan) [20]. Statistical significance was set at p < 0.05.

## 3 Results

### 3.1 Characteristics of study subjects

The characteristics of the study participants are listed in Table 1. There were 7,873 participants (3,304 men [42.0%] and 4569 women [58.0%]) with an average age of 71.7 years. The prevalence of insomnia symptoms was 23.4% in the overall population (21.9% in men and 24.5% in women). Compared to those without insomnia symptoms, the participants with insomnia symptoms showed a higher proportion of women; those experiencing difficult conditions on current income, pain/discomfort, anxiety, and frequent nocturia; those with longer time from bathing time to bedtime; and, those with bedroom lighting, and a lower prevalence of the feeling of happiness, lower BMI, and lower daily walking time. No significant differences were found in age or time spent using smartphones or other devices.

**Table 1:** Comparison of basic attributes and factors related to insomnia symptoms.

					
Variables		Total	AIS < 6 points	AIS ≥6 points	*P*-Value
					
Number (%)		7873 (100%)	6029 (76.6%)	1844 (23.4%)	
Sex	Male	3304 (42.0%)	2580 (42.8%)	724 (39.3%)	0.008
	Female	4569 (58.0%)	3449 (57.2%)	1120 (60.7%)	
Age	40–59	756 (9.6%)	568 (9.4%)	188 (10.2%)	0.784
	60–69	1739 (22.1%)	1330 (22.1%)	409 (22.2%)	
	70–79	4159 (52.8%)	3194 (53.0%)	962 (52.1%)	
	80–89	1222 (15.5%)	937 (15.5%)	285 (15.5%)	
BMI(kg/m^2^)	<18.5	517 (6.6%)	371 (6.2%)	146 (7.9%)	0.024
	18.5–24.9	5466 (69.4%)	4181 (69.3%)	1285 (69.7%)	
	25–29.9	1702 (21.6%)	1332 (22.1%)	370 (20.1%)	
	≥30	188 (2.4%)	145 (2.4%)	43 (2.3%)	
Living situation on current income	Very comfortable	108 (1.4%)	91 (1.5%)	17 (0.9%)	<0.001
	Somewhat comfortable	754 (9.6%)	609 (10.1%)	145 (7.9%)	
	Neutral	4647 (59.0%)	3713 (61.6%)	934 (50.7%)	
	Somewhat difficult	1905 (24.2%)	1339 (22.2%)	566 (30.7%)	
	Very difficult	459 (5.8%)	277 (4.6%)	182 (9.9%)	
Pain/discomfort	Yes	4631 (58.8%)	3223 (53.5%)	1408 (76.4%)	<0.001
	No	3242 (41.2%)	2806 (46.5%)	436 (23.6%)	
Anxiety	Yes	2296 (29.2%)	1284 (21.3%)	1012 (54.9%)	<0.001
	No	5577 (70.8%)	4745 (78.7%)	832 (45.1%)	
Feeling happiness	Yes	6771 (86.0%)	5435 (90.2%)	1336 (72.4%)	<0.001
	No	1102 (14.0%)	594 (9.8%)	508 (27.6%)	
Nocturnal urination frequency	0 times	1640 (20.8%)	1402 (23.3%)	238 (12.9%)	<0.001
	1 time	3276 (41.6%)	2605 (43.2%)	671 (36.4%)	
	2 times	2061 (26.2%)	1465 (24.3%)	596 (32.3%)	
	3 times	896 (11.4%)	557 (9.2%)	339 (18.4%)	
Time from bathing to bedtime (min)	<60	2253 (28.6%)	1825 (30.3%)	428 (23.2%)	<0.001
	60–119	3495 (44.4%)	2649 (43.9%)	846 (45.9%)	
	≥120	2125 (27.0%)	1555 (25.8%)	570 (30.9%)	
Bedroom lighting	No lighting	4696 (59.7%)	3645 (60.4%)	1051 (57.0%)	0.003
	Slightly Lighting	2511 (31.9%)	1902 (31.6%)	609 (33.0%)	
	Lighting	288 (3.7%)	198 (3.3%)	90 (4.9%)	
	Others	378 (4.8%)	284 (4.7%)	94 (5.1%)	
Time spent using smartphones, computers, and tablets	No use	1932 (24.6%)	1452 (24.1%)	480 (26.0%)	0.243
	<1 h	3608 (45.8%)	2803 (46.5%)	805 (43.7%)	
	1–2 h	1530 (19.4%)	1164 (19.4%)	366 (19.8%)	
	3–4 h	534 (6.8%)	402 (6.6%)	132 (7.2%)	
	≥4 h	269 (3.4%)	208 (3.4%)	61 (3.3%)	
Hours walked in a day	<30 min	1997 (25.4%)	550 (29.8%)	1447 (24.0%)	<0.001
	<1 h	2858 (36.3%)	686 (37.2%)	2172 (36.0%)	
	<2 h	2054 (26.1%)	417 (22.6%)	1637 (27.2%)	
	≥2 h	964 (12.2%)	191 (10.4%)	773 (12.8%)	

### 3.2 Factors associated with insomnia symptoms

Logistic regression analysis was performed to identify the factors associated with insomnia symptoms. In the simple analysis, the factors associated with insomnia symptoms were female sex, BMI <18.5, experiencing difficulty in living on current income, pain/discomfort, anxiety, lack of happiness, frequent nocturia, long time from bathing to bedtime, and short walking time **(**Table 2**)**. The multivariate logistic regression analysis showed that the factors independently associated with insomnia symptoms were age in the 80s (odds ratio [OR] 0.75, 95% confidence interval [Cl] 0.58–0.97, vs. 40–59 years), belonging to the female sex (OR 1.32, 95%Cl 1.16–1.50, vs. males), experiencing difficulty in living on current income (OR 1.63, 95%Cl 1.30–2.05, vs. neutral), having pain/discomfort (OR 1.90, 95%Cl 1.66–2.16) and anxiety (OR 3.21, 95%Cl 2.84–3.62), not feeling happiness (OR 2.03, 95%Cl 1.74–2.36), three times nocturia (OR 4.71, 95%Cl 3.73–5.94, vs. none), ≥120 min from bathing time to bedtime (OR 1.62, 95%Cl 1.39–1.90, vs. 60 min or less), bedroom lighting (OR 1.36, 95%Cl 1.02–1.81, vs. no lighting), ≥2 h daily walking (OR 0.70, 95% Cl 0.57–0.86, vs. < 30 min) **(**Table 2**)**. The Hosmer-Lemeshow goodness of fit test showed that the fitness of this model was acceptable (P = 0.09 > 0.05).

**Table 2:** Factors associated with insomnia symptoms.

Variables		Univariate analysis		Multivariate Analysis	
		Odd radio (95%CI)	*P*-Value	Odd radio (95%CI)	*P*-Value
Age	40–59	1.00		1.00	
	60–69	0.93 (0.76–1.13)	0.468	1.05 (0.84–1.31)	0.696
	70–79	0.91 (0.76–1.09)	0.304	0.97 (0.78–1.21)	0.800
	80–89	0.92 (0.74–1.14)	0.434	0.75 (0.58–0.97)	0.031
Sex	Male	1.00		1.00	
	Female	1.16 (1.04–1.29)	0.007	1.32 (1.16–1.50)	<0.001
BMI(kg/m^2^)	<18.5	1.28 (1.05–1.57)	0.016	1.15 (0.92–1.43)	0.230
	18.5–24.9	1.00		1.00	
	25–29.9	0.90 (0.79–1.03)	0.130	0.84 (0.73–0.98)	0.026
	≥30	0.96 (0.68–1.36)	0.840	0.69 (0.47–1.00)	0.052
Living situation on current income	Very comfortable	0.74 (0.44–1.25)	0.265	1.02 (0.59–1.77)	0.947
	Somewhat comfortable	0.95 (0.78–1.15)	0.580	1.04 (0.84–1.28)	0.725
	Neutral	1.00		1.00	
	Somewhat difficult	1.68 (1.49–1.90)	<0.001	1.29 (1.12–1.48)	<0.000
	Very difficult	2.61 (2.14–3.19)	<0.001	1.63 (1.30–2.05)	<0.001
Pain/discomfort	Yes	1.00		1.00	
	No	2.81 (2.50–3.17)	<0.001	1.90 (1.66–2.16)	<0.001
Anxiety	Yes	1.00		1.00	
	No	4.49 (4.02–5.02)	<0.001	3.21 (2.84–3.62)	<0.001
Feeling happiness	Yes	1.00		1.00	
	No	3.35 (3.05–3.97)	<0.001	2.03 (1.74–2.36)	<0.001
Nocturnal urination frequency	0 times	1.00		1.00	
	1 time	1.52 (1.29–1.78)	<0.001	1.69 (1.41–2.02)	<0.001
	2 times	2.40 (2.03–2.83)	<0.001	2.84 (2.33–3.45)	<0.001
	3 times	3.59 (2.96–4.35)	<0.001	4.71 (3.73–5.94)	<0.001
Time from bathing to bedtime (min)	<60	1.00		1.00	
	60–119	1.36 (1.19–1.55)	<0.001	1.39 (1.20–1.60)	<0.001
	≧120	1.56 (1.36–1.80)	<0.001	1.62 (1.39–1.90)	<0.001
Bedroom lighting	No lighting	1.00		1.00	
	Slightly Lighting	1.11 (0.99–1.24)	0.072	1.01 (0.89–1.15)	0.861
	Lighting	1.57 (1.22–2.04)	<0.001	1.36 (1.02–1.81)	0.035
	Others	1.15 (0.90–1.46)	0.266	0.99 (0.76–1.29)	0.928
Time spent using smartphones, computers, and tablets	No use	1.00		1.00	
	<1 h	0.87 (0.76–0.99)	0.033	0.97 (0.84–1.12)	0.674
	1–2 h	0.95 (0.81–1.11)	0.530	1.12 (0.93–1.34)	0.221
	3–4 h	0.99 (0.80–1.24)	0.953	1.11 (0.86–1.43)	0.437
	≥4 h	0.89 (0.65–1.20)	0.439	0.94 (0.66–1.33)	0.716
Hours walked in a day	<30 min	1.00		1.00	
	<1 h	0.83 (0.73–0.95)	0.005	0.96 (0.83–1.11)	0.616
	<2 h	0.67 (0.58–0.78)	<0.001	0.82 (0.70–0.96)	0.015
	≥2 h	0.65 (0.54–0.78)	<0.002	0.70 (0.57–0.86)	0.001

Subgroup analyses were conducted according to patient sex and age. By sex, factors associated only in women were BMI 25–29.9 (OR 0.79, 95%CI 0.64–0.96), BMI ≥30 (OR 0.51, 95%CI 0.31–0.84) (vs. BMI 18.5–24.9), bedroom lighting (OR 1.81, 95%CI 1.25–2.63, vs. no lighting), and 1–2 h use of smartphone/computer/tablet (OR 1.34, 95%CI 1.05–1.71, vs. no use). The factor associated with insomnia symptoms only in men was ≥2 h of daily walking (OR 0.63, 95% Cl 0.45–0.88, <30 min) (Supplementary file 1**)**. By age, factors associated only in those aged 65 years and older were 1–2 h from bathing to bedtime (OR 1.42, 95%CI 1.21–1.65, vs. <60 min); 1–2 h of daily walking time (OR 0.81, 95%CI 0.68–0.97); and, ≥2 h (OR 0.65, 95%CI 0.51–0.82, vs. <30 min). No associated factors were found in the patients aged <65 years (Supplementary file 2**)**.

## 4 Discussion

This study showed that insomnia symptoms was common (23.4%) in a Japanese community-based population, and three different factor groups, including physical (age, sex, BMI, pain/discomfort, frequent nocturia), psychological (anxiety, feeling happy), and environmental (living situation on current income, time from bathing time to bedtime, bedroom lighting, daily walking time) factors, were independently associated with insomnia symptoms. Furthermore, factors associated with insomnia symptoms varied according to sex and age.

### 4.1 Physical factors

The prevalence of insomnia symptoms was 10.0%–40.0% in previous studies [13,17,21], and it was higher, particularly in older people [22,23], women [24], obese subjects (BMI ≥30.0) [25], and those with pain and nocturia [7]. In the present study, the prevalence of insomnia symptoms was similar (23.4%) to that in other populations. Belonging to the female sex and having pain/discomfort and frequent nocturia were insomnia symptoms -related factors, which is in line with previous findings. Even a single nocturia episode was significantly associated with insomnia symptoms, and the more frequent the nocturia episodes, the higher the risk of insomnia symptoms. However, in contrast to previous findings, insomnia symptoms were less prevalent in the older population and in those with a high BMI, especially in women in the present study. Although the reason for this difference is unclear, insomnia symptoms may be associated with emaciation rather than obesity in this population.

### 4.2 Psychological factors

A previous study by Dragioti et al. showed that anxiety is strongly associated with insomnia symptoms in older adults [23]. Zhao et al. found that a decreased sense of well-being in older adults and women was associated with insomnia symptoms [8], and Otsuka et al. noted that subjective well-being in adolescence was strongly associated with the prevalence of sleep disorders [26]. Similarly, in the present study, 54.9% of patients with insomnia symptoms had anxiety, which was independently associated with insomnia symptoms, regardless of sex or age. Additionally, this study showed that happiness was independently associated with a decrease in insomnia symptoms. These results suggest that both positive and negative psychological factors are independently associated with insomnia symptoms.

Previous studies have reported that the majority of depressed subjects have insomnia and that depression and anxiety often co-exist [[27], [28], [29]]. Although this study could not assess depression due to the lack of questions on depression, depression is likely to co-occur with anxiety and may be related to sleep status.

### 4.3 Environmental factors

Regarding the association between income and insomnia symptoms, Lallukka et al. noted that a low socioeconomic status and household income were associated with insomnia [30]. The current study revealed that a difficult living condition in relation to current income was independently associated with insomnia symptoms. However, the group with comfortable living condition on current income did not show a significant decrease in the odds ratio for insomnia symptoms, suggesting that a worse living situation is associated with insomnia symptoms but an economically comfortable situation may not reduce insomnia symptoms.

Interestingly, the current study showed that the odds ratio of insomnia symptoms increased in subjects with a long time gap from bathing time to bedtime, especially in older people. Kalmbach et al. noted that exposure to stress interferes with sleep [31] and Harvey et al. noted that those with insomnia were more likely to worry about various things before bedtime [32], indicating that sleep is influenced by psychological factors [33]. Based on these findings, it may be recommended to avoid a long gap from bathing time to bed time to avoid factors that interfere with falling asleep easily.

Furthermore, the current study showed that the risk of insomnia symptoms was higher among those who slept in well-lit bedrooms than among those who slept in bedrooms with none or small night lights. Matsumoto et al. reported that lighting, rather than temperature or humidity, is the most relevant factor for insomnia [34] and Zeitzer et al. reported that melatonin production could be suppressed by normal room light [35]. It is reported that disruption of the sleep–wake rhythm can lead to insomnia [36]. Taken together, lighting conditions appear to be an important factor in the prevention of insomnia symptoms. Concerning the exercise, Chen et al. reported that walking improved sleep efficiency in older women with mild sleep disorders [37]. The current study also showed that insomnia symptoms were infrequent in the group that walked ≥2 h daily.

The subgroup analyses revealed that the factors associated with insomnia symptoms varied according to sex and age: BMI, bedroom lighting, smartphone use time for women, walking time for men, time between bathing and bedtime, and walking time for older people. This finding suggests that the advice for good sleep should be tailored to individual conditions rather than uniformity.

Combining previous reports and the results of the present study, it seems that not only drugs but also cognitive-behavioral therapy (CBT) from psychological and social environments may be effective intervention methods since these factors are related to sleep. Recently, CBT with telemedicine has been reported to be as effective as face-to-face intervention [38] and is expected to be an effective intervention method in the future.

### 4.4 Strengths and limitations of this study

The strength of this study is the robustness of the results owing to the large sample size that enables a more detailed analysis that includes various types of factors simultaneously and sub-grouped by age and gender. Consequently, this study simultaneously examined a wide range of insomnia symptoms-related factors individually reported in the literature. However, this study had several limitations. First, the study subjects were community-based health examination participants. In addition, the moderate response rate (69.7%) and the exclusion of those with missing data may have led to a possible selection bias, as relatively healthy individuals were included in the analysis. Secondly, sleep status is subjective and may differ from objective measurements. Third, because this was a cross-sectional study, it was not possible to determine the causal relationship. Fourth, the present study lacked information on the history of sleep-related illnesses and use of sleep-inducing medications.

## 5 Conclusions

This questionnaire-based study showed that insomnia symptoms is common in the Japanese community-based population, and three different factor groups are independently associated with insomnia symptoms. These results suggest that factors associated with insomnia symptoms may vary depending on each person's characteristics and situation and that measures tailored to the individual's condition might be required to improve insomnia symptoms.

## Ethics statement

The questionnaire package contained an overview of the study, including information on the voluntary nature of participation and protection of personal information. Written informed consent was obtained from all the participants. This study was conducted in accordance with the Declaration of Helsinki and approved by the Ethics Committee of Yamagata University School of Medicine (2009-1222, 2022-263).

## Funding

The Yamagata Cohort Study is supported by the 21st Century COE Program and Global COE Program.

## Data availability

The dataset of the current study was not publicly available for ethical reasons. However, it can be accessed by contacting the corresponding author upon reasonable request.

## Prior presentations

None.

## CRediT authorship contribution statement

**Miho Suzuki:** Writing – original draft. **Natsuko Suzuki:** Writing – review & editing. **Kaori Sakurada:** Writing – review & editing. **Norihiko Tsuchiya:** Writing – review & editing. **Yoshiyuki Ueno:** Writing – review & editing. **Tsuneo Konta:** Writing – review & editing.

## Declaration of competing interest

The authors declare that they have no known competing financial interests or personal relationships that could have appeared to influence the work reported in this paper.
