# Supplementary material for: Insomnia symptoms and related factors in a community-based population: The Yamagata Cohort study
Source: Heliyon. 2024 Mar 15;10(6):e28228. doi: 10.1016/j.heliyon.2024.e28228 (PMC10963640; doi:10.1016/j.heliyon.2024.e28228)
Supplement: Multimedia component 1 [file mmc1.docx]

| Supplementary Table 1.　Factors associated with insomniasymptoms（Male vs. Female） | | | | | | |
| --- | --- | --- | --- | --- | --- | --- |
| Variables |  | Male | |  | Female | |
|  |  | Odd radio (95%CI) | P-Value |  | Odd radio (95%CI) | P-Value |
| Age | 40-59 | 1.00 |  |  | 1.00 |  |
|  | 60-69 | 0.99 (0.63 - 1.55) | 0.972 |  | 0.91 (0.70 - 1.19) | 0.507 |
|  | 70-79 | 0.82 (0.54 - 1.25) | 0.359 |  | 0.86 (0.65 - 1.12) | 0.255 |
|  | 80-89 | 0.56 (0.35 - 0.90) | 0.017 |  | 0.69 (0.49 - 0.97) | 0.031 |
| BMI（kg/m^2^) | <18.5 | 0.87 (0.52 - 1.48) | 0.616 |  | 1.20 (0.93 - 1.54) | 0.153 |
|  | 18.5-24.9 | 1.00 |  |  | 1.00 |  |
|  | 25-29.9 | 0.87 (0.71 - 1.07) | 0.197 |  | 0.79 (0.64 - 0.96) | 0.021 |
|  | ≥30 | 0.95 (0.52 - 1.75) | 0.877 |  | 0.51 (0.31 - 0.84) | 0.008 |
| Living situation on current income | Very comfortable | 1.02 (0.34 - 3.01) | 0.973 |  | 0.98 (0.52 - 1.87) | 0.958 |
|  | Somewhat comfortable | 0.96 (0.67 - 1.37) | 0.806 |  | 1.09 (0.84 - 1.41) | 0.538 |
|  | Neutral | 1.00 |  |  | 1.00 |  |
|  | Somewhat difficult | 1.51 (1.22 - 1.86) | <0.001 |  | 1.13 (0.95 - 1.36) | 0.176 |
|  | Very difficult | 1.71 (1.19 - 2.44) | 0.003 |  | 1.55 (1.15 - 2.09) | 0.004 |
| Pain/discomfort | Yes | 1.00 |  |  | 1.00 |  |
|  | No | 2.01 (1.64 - 2.48) | <0.001 |  | 1.81 (1.53 - 2.15) | <0.001 |
| Anxiety | Yes | 1.00 |  |  | 1.00 |  |
|  | No | 3.10 (2.54 - 3.78) | <0.001 |  | 3.29 (2.81 - 3.83) | <0.001 |
| Feeling happiness | Yes | 1.00 |  |  | 1.00 |  |
|  | No | 1.92 (1.53 - 2.41) | <0.001 |  | 2.06 (1.67 - 2.53) | <0.001 |
| Nocturnal urination frequency | 0 times | 1.00 |  |  | 1.00 |  |
|  | 1 time | 1.67 (1.13 - 2.46) | 0.010 |  | 1.71 (1.40 - 2.10) | <0.001 |
|  | 2 times | 2.87 (1.94 - 4.26) | <0.001 |  | 2.85 (2.26 - 3.59) | <0.001 |
|  | 3 times | 4.68 (3.10 - 7.06) | <0.001 |  | 4.87 (3.49 - 6.81) | <0.001 |
| Time from bathing to bedtime (min) | <60 | 1.00 |  |  | 1.00 |  |
|  | 60 -119 | 1.48 (1.17 - 1.87) | 0.001 |  | 1.32 (1.10 - 1.58) | 0.003 |
|  | ≧120 | 1.43 (1.11 - 1.83) | 0.001 |  | 1.79 (1.46 - 2.20) | <.0001 |
| Bedroom lighting | No lighting | 1.00 |  |  | 1.00 |  |
|  | Slightly Lighting | 0.96 (0.79 - 1.18) | 0.713 |  | 1.02 (0.87 - 1.21) | 0.784 |
|  | Lighting | 0.88 (0.55 - 1.41) | 0.586 |  | 1.81 (1.25 - 2.63) | 0.002 |
|  | Others | 0.78 (0.50 - 1.23) | 0.280 |  | 1.12 (0.80 - 1.58) | 0.497 |
| Time spent using smartphones, computers, and tablets | No use | 1.00 |  |  | 1.00 |  |
|  | < 1 hour | 0.91 (0.72 - 1.14) | 0.396 |  | 1.06 (0.88 - 1.29) | 0.533 |
|  | 1-2 hours | 0.95 (0.71 - 1.26) | 0.702 |  | 1.34 (1.05 - 1.71) | 0.017 |
|  | 3-4 hours | 1.05 (0.73 - 1.53) | 0.782 |  | 1.21 (0.85 - 1.73) | 0.294 |
|  | ≥ 4 hours | 0.83 (0.50 - 1.38) | 0.481 |  | 1.16 (0.70 - 1.91) | 0.562 |
| Hours walked in a day | < 30 min | 1.00 |  |  | 1.00 |  |
|  | <1 hour | 0.94 (0.74 - 1.19) | 0.612 |  | 1.00 (0.83 - 1.20) | 0.983 |
|  | < 2 hours | 0.78 (0.60 - 1.01) | 0.061 |  | 0.86 (0.70 - 1.06) | 0.150 |
|  | ≥ 2 hours | 0.63 (0.45 - 0.88) | 0.006 |  | 0.77 (0.59 - 1.00) | 0.054 |

| Supplementary Table 2.　Factors associated with insomniasymptoms（Under 65 vs. over 65） | | | | | | |
| --- | --- | --- | --- | --- | --- | --- |
| Variables |  | Age＜65 years | |  | Age≥65 years | |
|  |  | Odd radio (95%CI) | P-Value |  | Odd radio (95%CI) | P-Value |
| Sex | Male | 1.00 |  |  | 1.00 |  |
|  | Female | 1.44 (1.02 - 2.02) | 0.038 |  | 1.44 (1.25 - 1.66) | <0.001 |
| BMI（kg/m^2^) | <18.5 | 1.16 (0.72 - 1.88) | 0.535 |  | 1.11 (0.86 - 1.43) | 0.420 |
|  | 18.5-24.9 | 1.00 |  |  | 1.00 |  |
|  | 25-29.9 | 1.02 (0.52 - 2.00) | 0.947 |  | 0.53 (0.33 - 0.86) | 0.010 |
|  | ≥30 | 0.67 (0.46 - 0.97) | 0.033 |  | 0.87 (0.74 - 1.02) | 0.083 |
| Living situation on current income | Very comfortable | 1.10 (0.33 - 3.68) | 0.882 |  | 0.98 (0.52 - 1.83) | 0.946 |
|  | Somewhat comfortable | 1.48 ( 0.91 - 2.41) | 0.116 |  | 0.96 (0.76 - 1.21) | 0.708 |
|  | Neutral | 1.00 |  |  | 1.00 |  |
|  | Somewhat difficult | 1.46 ( 1.06 - 2.02) | 0.020 |  | 1.28 (1.10 - 1.49) | 0.001 |
|  | Very difficult | 1.60 (0.99 - 2.57) | 0.054 |  | 1.68 (1.29 - 2.18) | <0.001 |
| Pain/discomfort | Yes | 1.00 |  |  | 1.00 |  |
|  | No | 1.71 (1.27 - 2.30) | <0.001 |  | 1.88 (1.62 - 2.17) | <0.001 |
| Anxiety | Yes | 1.00 |  |  | 1.00 |  |
|  | No | 3.30 (2.49 - 4.39) | <0.001 |  | 3.15 (2.76 - 3.61） | <0.001 |
| Feeling happiness | Yes | 1.00 |  |  | 1.00 |  |
|  | No | 2.70 (1.90 - 3.84) | <0.001 |  | 1.90 (1.61 - 2.26) | <0.001 |
| Nocturnal urination frequency | 0 times | 1.00 |  |  | 1.00 |  |
|  | 1 time | 1.77 (1.30 - 2.41) | 0.000 |  | 1.63 (1.31 - 2.03) | <0.001 |
|  | 2 times | 3.72 (2.42 - 5.71) | <0.001 |  | 2.60 (2.07 - 3.27) | <0.001 |
|  | 3 times | 5.70 (2.47 -13.1) | <0.001 |  | 4.21 (3.25 - 5.44) | <0.001 |
| Time from bathing to bedtime (min) | <60 | 1.00 |  |  | 1.00 |  |
|  | 60 -119 | 1.29 (0.89 - 1.88) | 0.180 |  | 1.42 (1.21 - 1.65) | <0.001 |
|  | ≧120 | 1.41 (0.94 - 2.13) | 0.100 |  | 1.68 (1.42 - 2.00) | <0.001 |
| Bedroom lighting | No lighting | 1.00 |  |  | 1.00 |  |
|  | Slightly Lighting | 0.83 (0.61 - 1.12) | 0.218 |  | 1.05 ( 0.91 - 1.20) | 0.521 |
|  | Lighting | 1.03 (0.49 - 2.16) | 0.937 |  | 1.36 ( 0.99 - 1.85) | 0.057 |
|  | Others | 0.82 (0.37 - 1.78) | 0.816 |  | 1.02 (0.76 - 1.36) | 0.903 |
| Time spent using smartphones, computers, and tablets | No use | 1.00 |  |  | 1.00 |  |
|  | < 1 hour | 1.26 (0.67 - 2.36) | 0.468 |  | 1.00 (0.86 - 1.16) | 0.982 |
|  | 1-2 hours | 1.40 (0.75 - 2.62) | 0.289 |  | 1.21(0.99 -147) | 0.057 |
|  | 3-4 hours | 1.51 (0.76 - 2.97) | 0.237 |  | 1.14 (0.84 - 1.54) | 0.397 |
|  | ≥ 4 hours | 1.42 (0.71 - 2.87) | 0.323 |  | 0.86 (0.50 - 1.47) | 0.573 |
| Hours walked in a day | < 30 min | 1.00 |  |  | 1.00 |  |
|  | <1 hour | 0.98 (0.68 - 1.41) | 0.901 |  | 0.98 (0.84 - 1.15) | 0.616 |
|  | < 2 hours | 0.95 (0.65 - 1.39) | 0.796 |  | 0.81 (0.68 - 0.97) | 0.015 |
|  | ≥ 2 hours | 1.08 (0.69 - 1.68) | 0.736 |  | 0.65 (0.51 - 0.82) | 0.001 |
